# Risk factors associated with in-hospital mortality during yellow fever outbreak in Brazil

**DOI:** 10.3389/fmed.2025.1505005

**Published:** 2025-01-27

**Authors:** Max McClure, Izabela Maurício de Rezende, Leonardo Soares Pereira, Maria Rita Teixeira Dutra, Jordana Rodrigues Barbosa Fradico, Rodrigo Macedo, Marcelle Cardoso Marçal, Lívia Soares Coelho Fonte Boa, Alexandre Maurício Castro Bragato, Flávio Augusto de Almeida Faria, Livia Pamplona, Rodrigo Fabiano do Carmo Said, Carlos Eduardo Calzavara-Silva, Dario Brock Ramalho, Cintia Lopes de Brito Magalhães, Pedro Augusto Alves, Thaysa Drummond Palmeira Gama, Gláucia Fernandes Cota, Thomas P. Monath, Olindo Assis Martins-Filho, Marcelo Antônio Pascoal-Xavier, Andrea Teixeira-Carvalho, Betânia Paiva Drumond, A. Desiree LaBeaud, Yellow Fever Collaborative Group, Argus Leão Araújo

**Affiliations:** ^1^Division of HIV, Infectious Diseases and Global Medicine, Department of Medicine, University of California, San Francisco, San Francisco, CA, United States; ^2^Division of Infectious Diseases, Department of Pediatrics, Stanford University School of Medicine, Stanford, CA, United States; ^3^Eduardo de Menezes Hospital, Belo Horizonte, Brazil; ^4^Integrated Group of Biomarkers Research, Instituto René Rachou, Fundação Oswaldo Cruz (Fiocruz), Belo Horizonte, Brazil; ^5^Pan American Health Organization, Brasília, Brazil; ^6^Cellular and Molecular Immunology, Instituto René Rachou, Fundação Oswaldo Cruz (Fiocruz), Belo Horizonte, Brazil; ^7^Research Center in Biological Sciences, Universidade Federal de Ouro Preto, Ouro Preto, Brazil; ^8^Immunology of Viral Diseases, Instituto René Rachou, Fundação Oswaldo Cruz (Fiocruz), Belo Horizonte, Brazil; ^9^Instituto René Rachou, Fundação Oswaldo Cruz (Fiocruz), Belo Horizonte, Brazil; ^10^Crozet BioPharma LLC, Lexington, MA, United States; ^11^Laboratory of Viruses, Microbiology Department, Biological Sciences Institute, Universidade Federal de Minas Gerais, Belo Horizonte, Brazil

**Keywords:** yellow fever, yellow fever virus, mortality, clinical management, Brazil

## Abstract

**Objective:**

To characterize the clinical manifestations of yellow fever disease and identify risk factors for mortality.

**Methods:**

A retrospective study was conducted in the referral center for infectious diseases (Hospital Eduardo de Menezes-HEM) in Belo Horizonte, Minas Gerais, Brazil. Analysis included data from 283 patients with confirmed YF infection older than 13 years old who presented to HEM between January 2017 and June 2018. In-hospital mortality (hypothesis formulated after data collection), demographic factors and clinical and laboratory assessments were used.

**Results:**

Study patients were mainly men (87.6%), with a median age of 46.0 (IQR 36.5, 57.0). 131 (46.3%) patients were admitted to the ICU, and 62 (22.0%) used invasive mechanical ventilation for a median of 2 days (IQR 1, 3). The median (IQR) total length of stay (LOS) in the ICU was 6 days (IQR 4, 8). The in-hospital mortality rate was 24.0%. Age was significantly higher in fatal (median 49.5, IQR 41.0, 61.0]) than in non-fatal cases [46 (36, 55)] (*p* < 0.01). Male sex was associated with an increased risk of death (RR 4.66, 95% CI 1.19, 18.2; *p* < 0.01). Most common symptoms and signs on admission to HEM were fever (31.9%), myalgia (27.8%), jaundice (24.3%), headache (23.9%), abdominal pain (16.1%), vomiting (12.2%), weakness (10.4%), and arthralgias (10.0%). Initial viral load above the cutoff of 4.45 log_10_ copies/mL was significantly associated with death prior to discharge (OR 12.2; CI 2.83, 92.3). Five factors were significantly related to increased odds of death prior to discharge: log-transformed AST (OR 3.65; CI 2.02, 7.81; *p* < 0.001), log-transformed INR (OR 7.40; CI 1.31, 33.0; *p* = 0.010), log-transformed lactate (OR 4.57; CI 1.48, 17.1; *p* = 0.013), log-transformed WBC (OR 4.33; CI 1.19, 18.5; *p* = 0.034), and age (OR 1.06; CI 1.01, 1.12; *p* = 0.026).

**Conclusions and relevance:**

AST, INR, lactate, WBC, and age are statistically associated with death prior to discharge in YF patients. These clinical markers should be applied to improve patient screening and management during future YF epidemics.

## 1 Introduction

Despite the existence of a safe yellow fever (YF) vaccine (17DD) since 1936 (1, 2), outbreaks in Angola (3), the Democratic Republic of Congo (4), and Brazil (5) have demonstrated that the causative agent of YF, yellow fever virus (YFV), is still a significant public health threat. Because the disease tends to occur in sporadic outbreaks in remote areas with limited access to medical care, its burden remains underestimated and its natural history is only partially characterized. The 2016-2018 YF outbreaks in southeastern Brazil offer an opportunity to better understand the disease’s clinical course.

YFV, the prototype virus of the *Flaviviridae* family (*Flavivirus* genus), is transmitted by mosquitoes of the *Haemagogus, Sabethes*, and *Aedes* genera (6, 7). Classically, the clinical syndrome comprises three phases: infection, marked by flu-like symptoms and viremia, accompanied by leukopenia and transaminitis; remission, characterized by seroconversion with the resolution of symptoms; and, in approximately 15% of patients, progression to intoxication, characterized by hemorrhagic fever, acute hepatitis, renal failure, and shock. This last phase has a mortality rate variously estimated at 20–50% (6, 8).

Following the intoxication phase, a few studies have recently described a late-relapsing hepatitis characterized by persistent fatigue and a rebound in aspartate aminotransferase (AST), alanine aminotransferase (ALT), total bilirubin (TBil) and alkaline phosphatase (ALP) within 6 months of an improvement or normalization of liver function (9–12). Commonly cited descriptions of the disease’s clinical course, however, are drawn from a highly variable set of cross-sectional and cohort studies (6, 13).

Although YF has been described for centuries in the Americas, previous outbreaks occurred in remote areas with low numbers of cases, making it difficult to study the disease. More recently, YF has spread throughout Angola, with 10–13% case fatality rate. (14), with imported cases being described in the Democratic Republic of Congo (14). The outbreak in Brazil in 2016–2018 was the largest in recent decades, causing 2,166 cases and 751 deaths (35% case fatality rate), primarily in the Southeast region. Minas Gerais (MG) state alone recorded 45% of cases, with 1,002 cases and 340 deaths (7, 15). In MG, the Hospital Eduardo de Menezes (HEM) was the referral hospital for YF patients in Belo Horizonte, MG, responsible for approximately 30% of the total cases in the state. Previous studies during this outbreak have showed that older age, elevated neutrophil count, increased AST, and higher viral load are associated with death in ICU patients (16, 17).

Using data from patients attended at HEM in 2017 and 2018, we retrospectively characterize the disease in this cohort and identify risk factors for mortality, including AST, INR, lactate, WBC, and age, that were associated with death prior to discharge in YF patients. We described clinical markers that should be applied to improve patient screening and management during future YF epidemics.

## 2 Materials and methods

### 2.1 Study design and data source

This study was conducted in a retrospective cohort, based on the review of inpatient medical records from a referral center for infectious diseases (Hospital Eduardo de Menezes – HEM) in Belo Horizonte, MG, during the 2017–2018 YF outbreaks in Brazil. Hospitalized patients met one or more of the Secretary of Health of Minas Gerais criteria for moderate (laboratory values of AST or ALT > 500 U/L, Creatinine > 1.3 mg/dL, vomiting, diarrhea or abdominal pain) or severe (laboratory values of AST or ALT > 2000 U/L, Creatinine > 2 mg/dL, RNI ≥ 1.5, platelets < 50,000/μL, oliguria, mental confusion, bleeding, breathing disorder (presence of dyspnea, oxygen requirement, or respiratory rate > 24 breaths per minute), diathesis, or jaundice) yellow fever (18). All patients with severe YF, based on the MoH classification, were admitted to the ICU. Patients were referred to HEM and hospitalized there after this initial screening, and a YFV PCR test was ordered for each patient. For patients who presented at the hospital with more than 6 days of symptoms, an ELISA test was also ordered (18).

### 2.2 Study population

Initially, analysis included patients older than 13 years old who presented to HEM between January 2017 and June 2018 with confirmed YF. YF diagnosis was performed by detecting IgM anti-YFV, YFV RNA by RT-qPCR or YFV isolation using serum samples. Patients diagnosed based on YFV IgM alone were only classified as confirmed YF if dengue IgM was negative, in order to exclude false positives due to cross-reactivity. Zika IgM was not required, but if detected the patient was reclassified as non-YF. Patients who presented after more than 21 days of symptoms were excluded. Patients who were transferred to or from another hospital were excluded from the final analysis, due to missing information on hospital course and final outcome.

### 2.3 Patient involvement

Our study was a retrospective data analysis and did not include patients as study participants. No patients were involved in setting the research question or the outcome measures, nor were they involved in the design and implementation of the study

### 2.4 Clinical outcomes

Clinical outcomes assessed included in-hospital mortality, ICU admission, use of invasive mechanical ventilation, total hospital length of stay (LOS), and ICU LOS. In-hospital mortality was defined as percentage of patients with confirmed YF who died in the hospital.

### 2.5 Ethical aspects

This study was approved by the Institutional Review Boards at Stanford University School of Medicine, under the eProtocol #53676, the Ethics committee at Instituto René Rachou (FIOCRUZ-MG), and Fundação Hospitalar do Estado de Minas Gerais (FHEMIG) under the protocols CAAE 72569317.2.0000.5091 and CAAE 65910317.0000.5071. No informed consent of study participants was pursued due to the nature of the deidentified data after IRBs’ authorization. This study followed the Strengthening the Reporting of Observational Studies in Epidemiology (STROBE) reporting guideline.

### 2.6 Yellow fever virus RNA detection and quantitation

Serial blood samples collected during the inpatient period were used for quantitative real-time reverse transcriptase followed by PCR (qRT-PCR). Briefly, YFV RNA was extracted from 140 μL of serum samples using the QIAmp Viral RNA Mini Kit (QIAGEN), following the manufacturer’s instructions. Total RNA (5 μL) was used in RT-qPCR targeting the 5’UTR region of the YFV genome (19). Positive samples were then used for quantitative qRT-PCR, using Bio Gene Research Yellow Fever PCR kit (Bioclin, Brazil), to determine the YFV RNA genomic viral load. The genomic viral load was expressed as log-transformed genomic copies (GC)/mL. The RNA quantification kit detects at least 20 GC/mL of viral RNA. For RNA quantification, the highest point for the standard curve (provided by the kit) was 2 × 10^5^ GC/mL and diluted up to 2 × 10^1^ GC/mL.

### 2.7 Clinical and demographic data

The demographic characteristics examined included age, sex, comorbid conditions, and YF immunization status. We also analyzed symptoms and signs that confirmed YF patients presented with at the time of admission as well as first recorded lab values, restricted to samples collected within hospital day three. Routine laboratory tests included blood counts, basic metabolic panels, liver and renal function tests, coagulation markers, blood gases, lactate, and additional inflammatory markers. The presence or absence of proteinuria during the hospitalization was analyzed separately, as the date of collection could not be verified for the majority of patients. Additional tests ordered according to individual providers’ judgment were ultrasonography, computed tomography, and bacterial cultures.

### 2.8 Statistical analysis

Data were maintained in the Stanford University REDCap platform. A descriptive analysis was performed to assess the distribution of patient demographic characteristics, hospital characteristics, clinical characteristics, medication use, and clinical outcomes by survival status (survived vs deceased). Continuous data were expressed as median (interquartile range [IQR]). Categorical variables were expressed as counts and percentages. We used χ2 or Fisher exact tests to evaluate statistical differences between groups for categorical variables and *t*-tests or Wilcoxon rank sum tests for continuous variables as appropriate. Relative risks by contingency table were calculated using unconditional maximum likelihood estimation with Wald confidence intervals. Odds ratios by contingency table were calculated using median-unbiased estimation with mid-p exact confidence intervals (20).

Multivariate logistic regression was used to calculate adjusted odds ratios for death prior to discharge. The maximal initial model was restricted to demographic features with *p* < 0.05 in the univariate analysis and initial laboratory values with *p* < 0.05 in the univariate analysis and greater than 250 observations to preserve sample size and avoid multicollinearity. Individuals missing any of the eligible covariates were excluded from analysis. Because a relatively small subset of participants provided information about symptoms or signs, these variables were not included in the multivariate analysis. Skewed variables were log-transformed. Direct bilirubin and lactate dehydrogenase were subsequently removed as predictors due to associated variance inflation factors (VIF) > 5 in the initial model. This initial screening process served to limit the number of candidate predictors, given the relatively small number of observations. An optimal model was then selected using a backward stepwise algorithm, which successively eliminates variables from the initial set of predictors until model fit no longer improves.

Analyses were conducted in Minitab 17 (Minitab Corp), MATLAB R2020a (Math-Works, Natick, Massachusetts) and R v4.3.1 (R Foundation for Statistical Computing, Vienna, Austria).

## 3 Results

**FIGURE 1:**
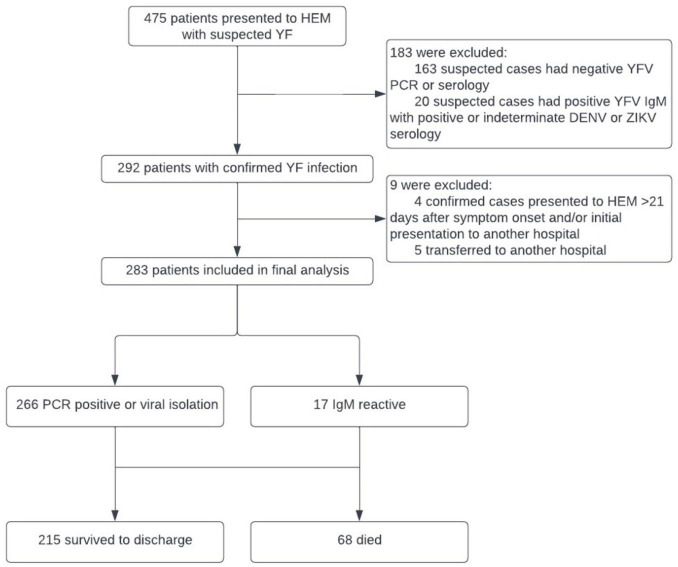
Cohort selection, based on yellow fever (YF) diagnosis, for analysis of risk factors for fatal and non-fatal disease.

During the YF outbreak in MG, 475 patients suspected of YF infection were referred to HEM from January 2017 to June 2018, of which 292 were confirmed (Figure 1). After excluding an additional 9 patients for presenting > 21 days after symptom onset or transferring prior to discharge, 283 patients were retained for analysis. 266 of these patients were diagnosed on the basis of PCR or viral isolation, and 17 were diagnosed using positive YFV IgM alone, followed by negative DENV IgM results.

Patients presented from a variety of municipalities across Minas Gerais state, with the highest case counts from municipalities near HEM (about within 150 kilometers distance) (Supplementary Figure 1). Patients meeting the YF criteria and included in the study population were mainly male (87.6%), with a median age of 46.0 (IQR 36.5, 57.0). Median delay between symptom onset and day of admission was 4 days (IQR 3, 6) and did not differ significantly between fatal (4 [3, 6]) and non-fatal cases (4 [3,6]). Of 282 patients for whom level of care was recorded, 131 (46.3%) were admitted to the ICU and 62 (22.0%) used invasive mechanical ventilation for a median of 2 days (IQR 1, 3). The median (IQR) total length of stay (LOS) in the ICU was 6 days (IQR 4, 8). The in-hospital mortality rate was 24.0% (Supplementary Figure 2).

**FIGURE 2:**
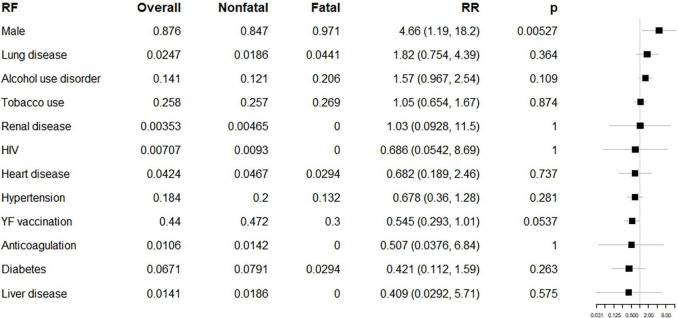
Epidemiologic risk factors (RF) for fatal and non-fatal cases. From left to right, columns list the RF of interest (in order of relative risk), its overall prevalence, prevalence among non-fatal cases, prevalence among fatal cases, relative risk with 95% confidence intervals, *p*-value by Fisher’s exact (uncorrected for multiple comparisons), and a forest plot on a logarithmic scale, illustrating the relative risk (node) and intervals (whiskers) relative to 1 (vertical line), where nodes to the right of the line indicate increased risk of fatal outcome. *p* < 0.00417 is significant using the Bonferroni correction.

Age was significantly higher in fatal (median 49.5, IQR 41.0, 61.0]) than in non-fatal cases by *t*-test (46 [36, 55]) (*p* = 0.005). Male sex was also associated with an increased risk of death (RR 4.66, 95% CI 1.19, 18.2; *p* = 0.005). Other medical comorbidities assessed on intake, including tobacco and alcohol use, were not significantly related to hospital outcomes (Figure 2).

Of the 218 YF patients for whom vaccination status was reported, 44.0% (96) had been previously vaccinated against YFV. Vaccination dates were missing for 4 vaccinated individuals. Including one patient with an incomplete vaccination date that may have fallen within the period of interest, 61 may have received vaccinations within 10 days of symptom onset. Including 5 patients with incomplete vaccination dates, 6 may have been vaccinated at an age less than 1 year. Six vaccinated patients were diagnosed with YF on the basis of serology without confirmatory serum qRT-PCR.

Considering all patients who received a vaccine at any point, there was a marginally significant protective effect for vaccination (RR 0.545, 95% CI 0.293, 1.01, *p* = 0.054). Discounting patients who may have received vaccinations within 10 days of symptom onset or under the age of 1, this effect disappears (RR 0.525, 95% CI 0.278, 1.88, *p* = 0.613). Potentially having received a vaccine within 10 days of symptom onset had no significant relationship to mortality (RR 2.03, 95% CI 0.459, 9.00, *p* = 0.486). Three YF patients had previously received two doses of the YFV vaccine, all of whom survived until discharge.

**FIGURE 3:**
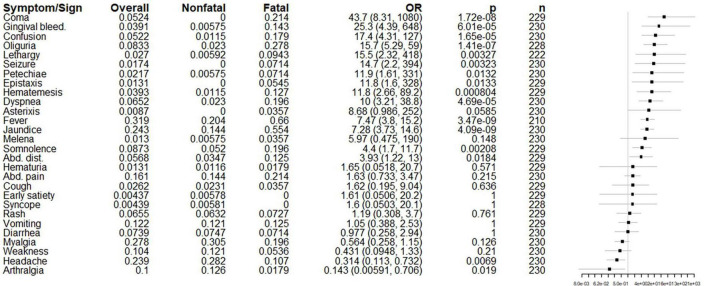
Initial symptoms and signs presented on the hospital admission of fatal and non-fatal cases. From left to right, columns list the risk factor of interest (in order of odds ratio), its overall prevalence, prevalence among non-fatal cases, prevalence among fatal cases, odds ratio with 95% confidence intervals, *p*-value by Fisher’s exact (uncorrected for multiple comparisons), the number of patients for which the presence or absence of each symptom was reported on admission, and a forest plot on a logarithmic scale illustrating the odds ratio (node) and intervals (whiskers) relative to 1 (vertical line), where nodes to the right of the line indicate increased odds of fatal outcome. *p* < 0.00179 is significant using the Bonferroni correction.

The most common symptoms and signs on admission to HEM were fever (31.9%), myalgia (27.8%), jaundice (24.3%), headache (23.9%), abdominal pain (16.1%), vomiting (12.2%), weakness (10.4%), and arthralgias (10.0%). After correcting for multiple comparisons, coma, gingival bleeding, fever, oliguria, confusion, jaundice, hematemesis, and dyspnea were significantly associated with fatal outcomes with *p*-values by Fisher’s exact less than 0.05. The number of patients asked about each symptom is listed in the accompanying figure (Figure 3).

**FIGURE 4:**
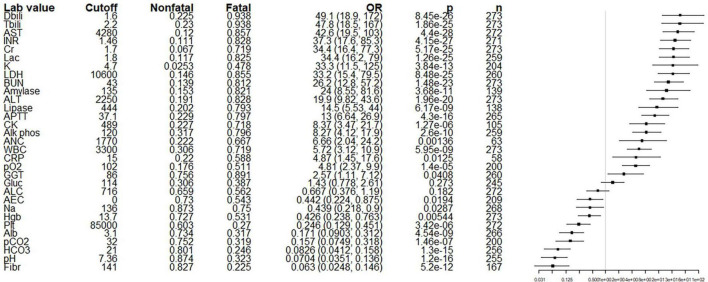
Initial basic laboratory values (at admission at HEM) of fatal and non-fatal cases. From left to right, columns list the risk factor of interest (in order of odds ratio), the cutoff value selected for dichotomization, the proportion of non-fatal cases above the cutoff, the proportion of fatal cases above the cutoff, odds ratio with 95% confidence intervals, *p*-value by Fisher’s exact (uncorrected for multiple comparisons), the number of patients for which the value was measured, and a forest plot on a logarithmic scale illustrating the odds ratio (node) and intervals (whiskers) relative to 1 (vertical line), where nodes to the right of the line indicate that a value above the cutoff is associated with a fatal outcome. *p* < 0.00161 is significant using the Bonferroni correction. Alb, albumin; ALC, absolute lymphocyte count; ALP, alkaline phosphatase; ALT, alanine aminotransferase; aPTT, activated partial thromboplastin time; AST, aspartate aminotransferase; BUN, blood urea nitrogen; CK, creatinine kinase; Cr, creatinine; CRP, C-reactive protein; DBili, direct bilirubin, Fibr, fibrinogen; INR, international normalized ratio; Lac, lactate; LDH, lactate dehydrogenase; pCO_2_, partial pressure of CO_2_; Plt, platelets; Tbili, total bilirubin; WBC, white blood cell count. Units are mg/dL for TBili, Dbili, BUN, Cr, and Fibr; U/L for AST, Amylase, LDH, Lipase, ALT, ALP and CK; mmol/L for Lac; sec for aPTT; mEq/L for K and HCO3; mg/L for CRP; mmHg for pO2 and pCO2, g/dL for Alb; Abd, abdominal; RP, retroperitoneal; schisto, schistosomiasis; irreg, irregular; GB, gallbladder; CBD, common bile duct; dil, dilatation; LAD, lymphadenopathy; intrahep, intrahepatic; corticomed diff, corticomedullary differentiation.

Basic laboratory results on presentation with *p*-values < 0.05 after Bonferroni correction included markers potentially associated with liver injury (elevations in bilirubin, transaminases, alkaline phosphatase, LDH, and markers of coagulopathy), kidney injury (elevations in BUN, creatinine, and potassium), lactic acidosis (elevated lactate and decreased pH, partial pressure of carbon dioxide, and serum bicarbonate), and other markers of severe illness (elevated amylase, lipase, creatinine kinase and white blood cell and neutrophil counts, and low albumin and partial pressure of oxygen) (Figure 4 and Supplementary Table 1). Many of these markers are non-specific and could reflect multiple processes (e.g., LDH, BUN, potassium), and all could reflect multiple organ dysfunction rather than injury to an isolated organ system. Proteinuria was present at any point during the hospitalization in 43.0% of individuals for whom the value was reported (*n* = 149) and was also significantly associated with death prior to discharge (OR 5.61, 95% CI 2.05, 18.4, *p* = 0.0005).

Of findings identified on abdominal, renal and lung ultrasound exams conducted at any point during subjects’ inpatient courses, only ascites (OR 15.3 [5.54, 48.5]) and loss of renal corticomedullary differentiation (9.62 [3.14, 32.5]) have *p*-values by Fisher’s exact less than 0.05 with Bonferroni correction (Supplementary Figure 3).

After accounting for likely contaminants, at least one positive blood culture was reported in 9.0% of the 222 YF patients from whom cultures were collected, with *E. coli* the most common isolated species (Supplementary Table 2). Positive blood cultures were significantly associated with mortality (OR 7.93 [95% CI 2.97, 23.9], *p* = 2.47 × 10^–5^). The respiratory tract was the most common suspected source of infections overall (19/33 infections for which a site was reported) (Supplementary Table 3).

Tested serum samples were positive for the presence of YFV RNA from 1 to 26 days post symptom (DPS) onset. Among samples on which we performed YFV RNA quantification, RNA YFV viral load varied from 15.75 GC/mL to 5.8 × 10^9^ GC/mL (*n* = 91).

The median time to peak of YFV viral load was 5 DPS (IQR 4, 6). Initial log_10_-transformed viral load (ranging from DPS 1 to 13) varied significantly between fatal and non-fatal cases by *t*-test (*p* = 0.01) (Supplementary Figure 4). Log_10_-transformed viral load remained significantly higher in fatal cases from days 2–3 and 5–7 through day 9 by Wilcoxon rank sum test using the Bonferroni correction (Supplementary Figure 5). After dichotomizing, an initial viral load > 4.45 log_10_ copies/ml was significantly associated with death prior to discharge (OR 12.2, 95% CI 2.83, 92.3, *p* = 0.0004).

In the final multivariate logistic regression model (using 212 complete cases), five factors were significantly related to an increased odds of death prior to discharge: log-transformed AST, log-transformed INR, log-transformed lactate, log-transformed WBC, and age. Sex was marginally significant (OR 4.22, 95% CI 0.272, 9.06, *p* = 0.0979) but was included in the final model as a potential confounder and because it improved model performance by AIC (Table 1). Likelihood ratio Chi-squared statistic relative to the null model was 162.93 (*p* = 2.2 × 10^–16^). The Hosmer-Lemeshow test was not significant, indicating reasonable fit.

**TABLE 1:** Odds ratios, confidence intervals and associated *p*-values for the selected multivariate model.

Variable	OR	CI	*p*-value
Sex	67.7	1.31, 8600	0.0979
Age	1.06	1.01, 1.12	0.0259
Log (AST)	3.65	2.02, 7.81	0.000128
Log (WBC)	4.33	1.19, 18.5	0.0335
Log (INR)	7.40	1.31, 33.0	0.00974
Log (Lac)	4.57	1.48, 17.1	0.0132

Log indicates natural log transformation. AST, aspartate aminotransferase; WBC, white blood cell count; INR,: international normalized ratio; Lac, lactate.

## 4 Discussion

Recent outbreaks in southeastern Brazil have highlighted the relatively limited clinical data available for predicting disease progression in YF patients. Results from our clinical dataset suggest that a number of demographic factors and laboratory values available at the time of admission – AST, INR, lactate, WBC, and age – are statistically associated with death prior to discharge. Our conclusions pertain to YFV genotype South American I virus, the causative pathogen of the 2017–2018 outbreaks (21).

In the multivariate analysis, age was significantly higher in fatal than in non-fatal cases, agreeing with prior studies: significantly higher mortality rates have been observed for patients older than 30 [Nigeria (22) and Ghana (23)] and older than 45 [Brazil (16)], and significantly lower median ages reported among non-fatal as compared to fatal cases [25 vs 31, Brazil (24); 37 vs 55, Brazil (17)]. Although many early observers of the disease believed that young adults faced higher risk not only of contracting the disease but worse outcomes once infected (25), this relationship likely reflects the age composition of those exposed to YFV-infected mosquitoes at the time. Studies with YFV-17D vaccine strain have shown that elderly people (≥60 years) were slower to develop an antibody response and had higher YF viremia levels than younger persons (26), which could explain the worse outcomes among old adults during natural infection.

A relationship between male sex and death before discharge, which was included in the multivariate model but did not reach significance, is also consistent with others’ findings (16, 27). Men are historically overrepresented in YF cases in South America due to occupational risk factors (28), but the explanation for a higher case fatality rate is less clear. It is also known that sex differences could solely interfere with the immune response, resulting in sex-specific outcomes from infectious diseases (29). In one clinical trial of YFV-17D vaccines, antibody levels were higher in males than females, suggesting more active viral replication (26, 30).

Most of the initial laboratory results associated with fatal disease in our study – AST, INR, and lactate – are consistent with the acute liver injury, lactic acidosis and generalized inflammation known to accompany YF. Transaminases have typically been elevated in all or virtually all patients in most YF outbreaks (31–33) with AST, ALT, and direct bilirubin found to be significantly higher among fatal cases in multiple studies (16, 17, 24). Prolonged prothrombin time (PT) is also common (32, 34) with an elevated PT significantly associated with increased mortality or significantly elevated in fatal cases (16, 35), as is thrombocytopenia (17). Stage 3 acute kidney injury and markers of lactic acidosis were significantly associated with increased mortality in one ICU study (35).

It is less clear if an elevated white blood cell count, which was also associated with mortality in multivariate analysis in our study, is a reliable indicator of disease severity. Early studies note both leukopenia and leukocytosis (22, 33, 36, 37) as a disease severity marker. More recently, higher white blood cell counts (17) and higher absolute neutrophil counts (16) have been significantly associated with mortality (though notably no patient in the latter study had frank neutrophilia), while leukopenia has been associated with lower mortality (35). Overall, these results suggest that markers of liver injury and sepsis that are already monitored by clinicians in the course of managing acute hepatitis may also have prognostic significance, as increases in transaminases, INR, lactate, and white blood cell count are all associated with increased risk of dying while inpatient.

YFV VL was omitted from our multivariate model due to small sample size. Nevertheless, in our univariate analysis, we found that an initial VL above the cutoff of 4.45 log_10_ copies/mL was significantly associated with death prior to discharge. This threshold is lower than that previously reported – Kallas et al. (16, 21) find a cutoff of 5.1 log_10_ copies/mL to be associated with death, though this study analyzed only patients that were in an intensive clinical unit, while in our study we analyzed everyone attended at HEM during the YF outbreak in 2017–2018. This difference in patient populations could explain the disparity in VL cutoffs, as critical cases might be expected to present with higher VL. Because high VL is usually linked to severe patients, it could also indicate a candidate for off-label sofosbuvir treatment, as previously described (38). Differences may also be attributable to approaches in defining threshold optimality. VL appears to remain notably higher in fatal cases through DPS 9, suggesting that VL may remain useful as a predictor of severe disease even in late-presenting patients. Including a VL test in the YF patient routine could improve their clinical management.

While health authorities focus on molecular diagnosis through 10 DPS (39), we detected YFV RNA in serum samples until 26 DPS. Persistence of YFV RNA has previously been demonstrated in serum and urine (40–42) of YF patients. When serological testing alone may not be sufficient to resolve the diagnosis, as in hyper-epidemic sites for dengue where they may be a cross reaction between anti-DENV and anti-YFV antibodies (43), combining the serological test with PCR even in more advanced days of the infection would likely contribute to more accurate diagnosis.

Additionally, although VL generally declined from approximately DPS 4 through 10, both fatal and non-fatal cases registered a rise in VL from approximately 12 through 15 DPS. The timing of this rise corresponds roughly to the appearance of YF antibodies, as discussed above in relation to YF vaccination status. While this finding could simply represent noise in the time series, it could also reflect antibody-dependent enhancement (ADE) of YFV replication. ADE leading to YFV viremia has been suggested before: after vaccination by a recombinant live attenuated chimeric YF and Japanese encephalitis vaccine, YF immune subjects were noted to have higher levels of viremia than YF non-immune subjects, though sample sizes were too small to perform hypothesis testing (44). However, we are unable to fully evaluate this possibility with our dataset.

Otherwise, our analysis of reported symptoms, ultrasound findings and superinfections were restricted to univariate analysis. Coma, hemorrhagic diathesis, fever, oliguria, confusion, jaundice and dyspnea– all expected features of severe disease – were associated with death prior to discharge. Prior data on clinical predictors of disease severity are limited: early studies conducted minimal or no statistical analyses on the prognostic significance of the clinical manifestations of YF, but authors suggest that poor prognostic signs are rigors, Faget’s sign, and decreased appetite (22) hematemesis, anuria, early jaundice, coma and preceding delirium(36), or bleeding diathesis of any kind (37). More recent studies have found significant associations between mortality and jaundice (17, 24), seizures and coma (35), or, in one case, no significant associations between any clinical finding and disease outcome (16). Our ultrasound findings and culture results are non-specific and reflective of critical illness, though sonographic changes in renal cortex echogenicity in particular were shown to be significantly associated with 30-day mortality in one study of yellow fever patients (45).

Of note, the number of patients previously vaccinated against YF in this cohort (*n* = 96) is high and encompasses patients with a wide range of intervals between vaccination date and symptom onset. For lifelong protection against YF, for children older than 9 months to adults up to 59 years, a single dose of YF vaccine has been recommended (46). As of a 2013 SAGE Working Group report on the YF vaccine (47), only 12 confirmed cases of breakthrough YF had been identified since the introduction of the vaccine. Our group had previously studied adverse events following YF vaccination (48) using the Brazil Ministry of Health case definition (49). In the referred study, none of the analyzed and sequenced possible cases were grouped within the YFV vaccine genotype, showing that all cases were due to natural YF infection (48).

There were several possible mitigating factors for a subset of apparent vaccine failures. Six of the 96 previously vaccinated patients were diagnosed on the basis of serology alone, which could potentially represent misclassification due to antibodies that persisted from the time of vaccination. The remaining cases (*n* = 90) were diagnosed based on qRT-PCR and were therefore highly unlikely to reflect vaccine failures due to waning immunity or adverse events after vaccination (48–50).

Six of the 96 previously vaccinated patients potentially received the YF-17D dose before 1 year of age. Several clinical trials of both YF-17D and YF-17DD vaccines have reported lower seroconversion rates in children ranging from ages 9 to 23 months (51) relative to those seen in adults, though a subsequent systematic review of pediatric data found no difference in seroconversion rates between children below and above the age of 9 months (52). 61 participants also reported a possible short (<10 days) interval between vaccination and symptom onset. International Health Regulations do not consider protective immunity against YFV to be present until 10 days after YF vaccine administration, (53) a recommendation based on multiple studies showing 80% or greater seroconversion starting day 10–14 after immunization (54–56). Our ability to fully filter out patients that fell into one of these categories was limited by incomplete information on patients’ vaccination dates and by the self-reported nature of many patients’ vaccination history. It is important to note that our interpretation of this part of the study could be biased due to a lack of information on the patient’s vaccination record and that the YF vaccine continues to be safe, and the best strategy against YF.

Our study is ultimately limited by virtue of being a retrospective analysis, with a lack of standardization in the clinical approach and variation in the data available for each individual. The data reflect the experience of a single hospital in a single state in Brazil, although it is noteworthy that HEM received about 30% of the total YF cases of MG. The study was conducted on a convenience sample of patients presenting to HEM, which may not be representative of the Brazilian (or Minas Gerais) population as a whole. While disease prevalence is roughly similar between our study population and that of Minas Gerais in 2017 for diabetes (6.7% vs. 5.6%) and HIV (0.71% vs. 0.31%), rates of reported alcohol use disorder (14.1% vs 3.98%), chronic kidney disease (0.35% vs 8.6%) and chronic liver disease (1.4% vs 24.15%) are widely divergent, reflecting either the non-representative nature of the sample or incomplete medical histories provided by study participants) (57). The in-hospital mortality of 24% we observed is also notably lower than both the 47% case fatality rate reported in a systematic analysis of other outbreaks (27) and the 36–44.2% reported in contemporary Brazilian cohorts (16, 24). It is possible that our exclusion of individuals who were transferred to or from HEM and/or who presented very late in their disease course could bias our assessment of in-hospital outcomes, but the overall effect on disease severity in our sample is not clear. Excluding individuals who presented > 21 days after symptom onset is likely to exclude milder cases that were no longer at risk for the intoxication phase. Transfers to and from HEM occur for a variety of reasons, potentially including disease severity.

Data collected during the study also varied widely between participants, guided by individual clinicians’ judgment, which may affect the reliability of statistical analysis. In particular, because symptoms and signs were reported for a relatively small subset of individuals, we are unable to say whether these data are representative of the cohort as a whole. Finally, because we did not test our cohort for IgG directed against other flaviviruses, we are unable to comment on whether prior Zika or Dengue infection affects survival. Some studies have shown that prior infection with a heterologous flavivirus protects against severe or fatal yellow fever both *in vivo* experiments (58, 59) and in cohort studies (60).

Within these constraints, our clinical and laboratory indicators of severe disease are broadly similar to those reported in other studies (16, 24, 32) and to the clinical signs of severity highlighted in the Minas Gerais Secretary of Health’s official recommendations for the clinical management of YF (oliguria, somnolence, confusion, coma, seizures, bleeding, respiratory difficulty, hypotension, signs of hypoperfusion, transaminases > 2000 IU/mL, creatinine > 2 mg/dL, INR > 1,5, and/or platelet count < 50000) (18), as well as to general markers of shock. As YF qRT-PCR becomes more widely available, our data suggest that this test may also be useful for early prognostication. Because standard of care for YF is supportive (61), early identification of high-risk cases that may require ICU-level care plays a primary role in management algorithms. Data derived from this, and other clinical cohorts may improve triage and direct critical care resources during future epidemics.

## Data Availability

The original contributions presented in this study are included in this article/Supplementary material, further inquiries can be directed to the corresponding author.

## Yellow Fever Collaborative Group Members

Argus Leão Araújo, Barbara Lenoir, Bruno Dala Vedova Gomes Beato, Carolina Lins Rodrigues Vieira, Daniel Vitor de Vasconcelos Santos, Flavia Mansur Starling, Gabriela Miana de Mattos Paixão, Indiara Penido, Izabela Aparecida Coelho, Leandro Henrique Malta Cunha, Letícia Menezes, Livia Frota Rabelo, Letícia Lemos Jardim, Lívia Fulgêncio da Cunha Melo, Lívia Zignago Moreira dos Santos, Ludmila de Paula, Luísa Lages de Abre Paladino, Natalia Soares Albuquerque, Simone Lopes Oliveira Lemos, Tayrine Araújo.
