# Supplementary material for: Risk factors associated with in-hospital mortality during yellow fever outbreak in Brazil
Source: Front Med (Lausanne). 2025 Jan 27;12:1505005. doi: 10.3389/fmed.2025.1505005 (PMC11807806; doi:10.3389/fmed.2025.1505005)
Supplement: Supplementary file 1 [file Data_Sheet_1.docx]

Supplementary Material

# Supplementary Figures


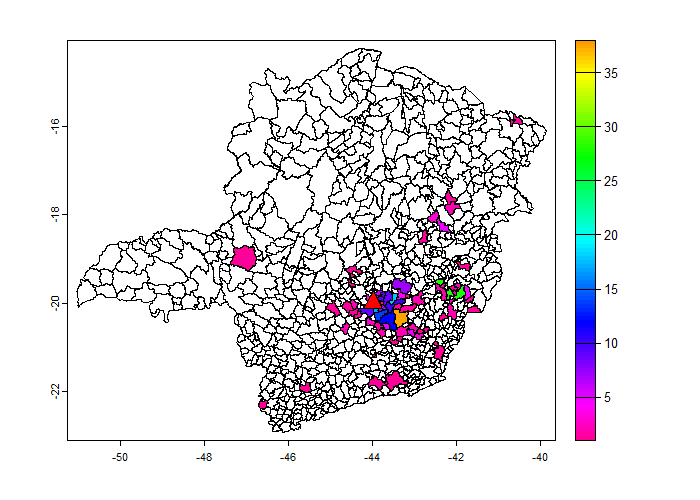


**Supplementary Figure 1.** YF cases treated at HEM, 2017-2018 – Municipality of origin for YF cases in the present study. Municipalities in the state of Minas Gerais are colored according to the number of confirmed YF cases attended at HEM as indicated by the legend. Municipalities without any confirmed YF cases that were attended at HEM are shown in white. The location of HEM is indicated by the red triangle.


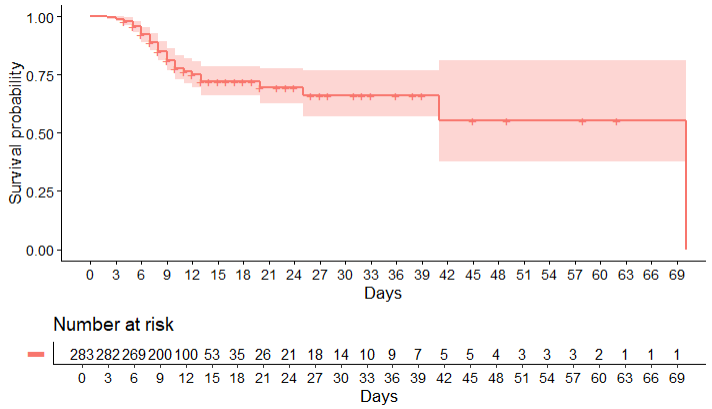


**Supplementary Figure 2.** Kaplan-Meier estimator for all inpatient YF cases at HEM, 2017-2018. The y axis indicates the proportion of patients alive, and the x axis indicates the number of days post symptoms (dps) Shaded areas represent 95% confidence intervals. The risk table below the plot tabulates the sample size at each timepoint. Discharged patients are censored.


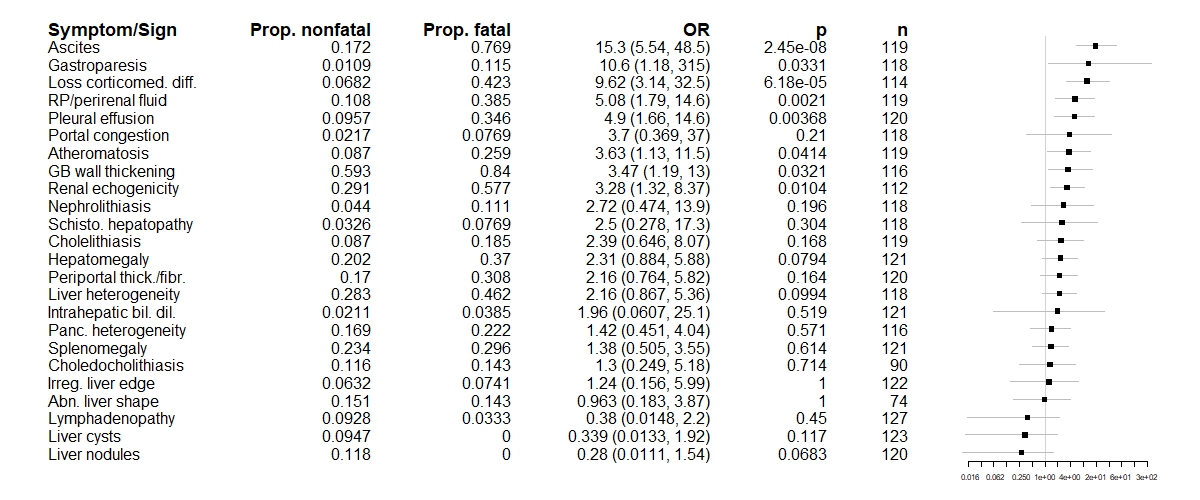


**Supplementary Figure 3.** Ultrasound findings of fatal and nonfatal cases. From left to right, columns list the risk factor of interest (in order of odds ratio), its overall prevalence, prevalence among nonfatal cases, prevalence among fatal cases, odds ratio with 95% confidence intervals, p value by Fisher’s exact (uncorrected for multiple comparisons), sample size, and a forest plot on a logarithmic scale illustrating the odds ratio (node) and intervals (whiskers) relative to 1 (vertical line), where nodes to the right of the line indicate increased odds of fatal outcome. p<0.00208 is significant using the Bonferroni correction.


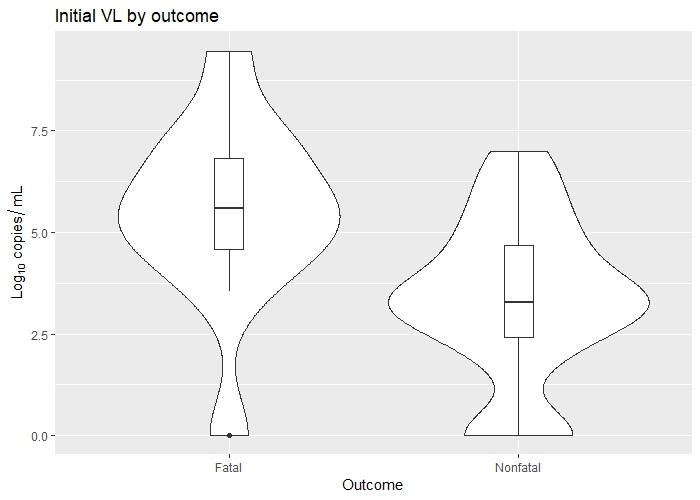


**Supplementary Figure 4.** Initial log_10_-transformed viral load by outcome shown as violin plot and superimposed boxplot. Marker indicating median value, hinges indicating quartiles, and whiskers indicating range.


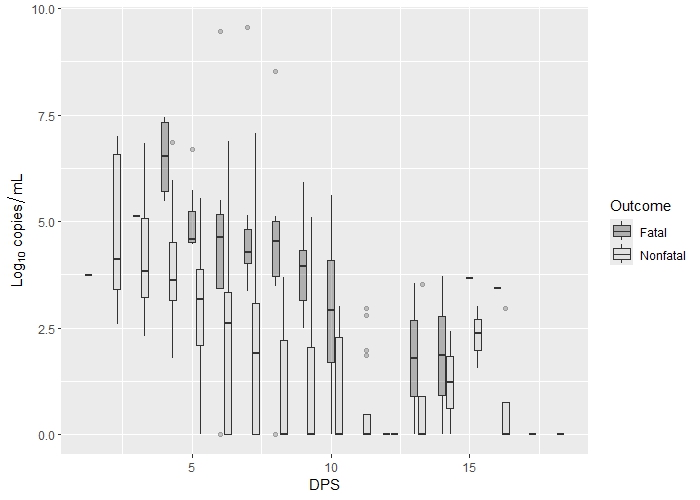


**Supplementary Figure 5.** Log_10_-transformed viral load for fatal and nonfatal cases by days post-symptom onset. Marker indicating median value, hinges indicating quartiles, and whiskers indicating range.

**Supplementary Table 1**: Median initial laboratory values (at admission day at HEM) and interquartile ranges for fatal and nonfatal cases, with p values by Wilcoxon rank sum test.

| Value | Overall median | Overall IQR | Nonfatal median | Nonfatal IQR | Fatal median | Fatal IQR | Wilcoxon |
| --- | --- | --- | --- | --- | --- | --- | --- |
| AEC | 27 | 0, 60 | 28 | 0, 59 | 23 | 0, 60.25 | 0.302906914 |
| Alb | 3.4 | 3, 3.8 | 3.5 | 3.1, 3.9 | 2.9 | 2.5, 3.25 | 3.52759E-09 |
| ALC | 921 | 601.5, 1322.5 | 956 | 614.25, 1302.75 | 863 | 560.25, 1545 | 0.567045989 |
| Alk phos | 109 | 76.5, 158 | 98 | 73, 135 | 161.5 | 127, 200.25 | 5.03014E-09 |
| ALT | 1235 | 434, 3194 | 726 | 318, 1807 | 4327.5 | 3029, 5901.75 | 6.74825E-21 |
| Amylase | 98 | 72, 146.5 | 89 | 69.5, 123 | 227.5 | 161.25, 505.5 | 1.22876E-09 |
| ANC | 1460 | 1002.5, 2241 | 1310 | 840, 1701 | 2522 | 1537.75, 4096.75 | 0.001042179 |
| APTT | 32.4 | 24.4, 43.9 | 28.3 | 23.3, 36.4 | 50.9 | 39.975, 77.025 | 4.48818E-17 |
| AST | 1440.5 | 372.5, 6361.5 | 724 | 308, 2289 | 8605 | 6900, 15179 | 1.21561E-24 |
| BUN | 31 | 22, 55.1 | 26.1 | 20.3, 37.1 | 94.6 | 49.925, 132.325 | 1.6191E-21 |
| CK | 303 | 122, 923 | 181 | 83, 359.5 | 858 | 408, 1512.5 | 5.91846E-07 |
| Cr | 0.9 | 0.7, 1.4 | 0.8 | 0.7, 1 | 4.1 | 1.375, 5.85 | 6.92762E-19 |
| CRP | 10 | 5, 20 | 6 | 4, 13 | 19 | 11, 31 | 0.001255172 |
| Dbili | 0.8 | 0.4, 3.8 | 0.5 | 0.3, 1.4 | 6.05 | 3.575, 7.525 | 1.7335E-24 |
| Fibr | 164 | 125, 212 | 186 | 150, 233 | 109 | 80.25, 138.75 | 2.63926E-12 |
| GGT | 192.5 | 99.75, 343.25 | 165 | 90, 344 | 220 | 137, 285.5 | 0.180949764 |
| Gluc | 101 | 87, 126 | 100 | 87.5, 125 | 106.5 | 86.25, 134.5 | 0.409747204 |
| HCO3 | 23 | 20, 25 | 24 | 22, 26 | 16 | 11, 21 | 3.21661E-14 |
| Hgb | 14.6 | 13.4, 15.8 | 14.7 | 13.6, 15.8 | 13.95 | 12.7, 15.325 | 0.018408571 |
| INR | 1.1 | 1, 1.515 | 1.02 | 1, 1.2 | 2.49 | 1.5175, 3.765 | 6.22176E-26 |
| K | 3.9 | 3.6, 4.3 | 3.9 | 3.6, 4.1 | 4.7 | 3.825, 5.2 | 1.82852E-06 |
| Lac | 1.2 | 1, 2.05 | 1.1 | 0.9, 1.425 | 3.4 | 2, 7.35 | 1.22037E-22 |
| LDH | 3719 | 1320.25, 15688.75 | 2114.5 | 1054.25, 6792.25 | 33750 | 18188.75, 45000 | 2.10955E-23 |
| Lipase | 300.5 | 161.75, 556.75 | 256 | 147, 402 | 973 | 459, 5145 | 1.49244E-08 |
| Na | 141 | 138, 143 | 141 | 138, 143 | 140.5 | 136.75, 143 | 0.20429185 |
| pCO2 | 36 | 31, 40 | 37 | 33, 40 | 30 | 25, 37 | 5.36988E-06 |
| pH | 7.41 | 7.36, 7.44 | 7.42 | 7.39, 7.44 | 7.33 | 7.23, 7.38 | 8.79512E-15 |
| Plt | 89000 | 63750, 122250 | 93000 | 69000, 131000 | 71000 | 46000, 88500 | 5.4883E-06 |
| pO2 | 81 | 54.75, 103 | 75 | 50, 97 | 103 | 76, 139 | 4.09919E-06 |
| Tbili | 1.2 | 0.6, 4.4 | 0.8 | 0.5, 1.8 | 6.7 | 4.2, 8.3 | 3.58906E-24 |
| WBC | 2900 | 2300, 4700 | 2700 | 2200, 3600 | 5450 | 3275, 9050 | 2.7327E-10 |

Legend: AEC: absolute eosinophil count; Alb: albumin; ALC: absolute lymphocyte count; ALP: alkaline phosphatase; ALT: alanine aminotransferase; ANC: absolute neutrophil count; aPTT: activated partial thromboplastin time; AST: aspartate aminotransferase; BUN: blood urea nitrogen; CK: creatinine kinase; Cr: creatinine; CRP: C-reactive protein; DBili: direct bilirubin, Fibr: fibrinogen; GGT: gammaglutamyl transpeptidase; Gluc: glucose; Hct: hematocrit; Hgb: hemoglobin; INR: international normalized ratio; Lac: lactate; LDH: lactate dehydrogenase; pCO_2_: partial pressure of CO_2_; Plt: platelets; pO_2_: partial pressure of O_2_; Tbili: total bilirubin; WBC: white blood cell count. Units are mg/dL for TBili, Dbili, BUN, Cr, and Fibr; U/L for AST, Amylase, LDH, Lipase, ALT, ALP, CK and GGT; mmol/L for Lac and Gluc; sec for aPTT; mEq/L for K, HCO3 and Na; mg/L for CRP; mmHg for pO2 and pCO2, g/dL for Alb and Hgb.

**Supplementary Table 2:** Species isolated from blood cultures of YF patients at any point during hospitalization. Coinfections are reported as if they were individual positive cultures.

| Spp | Count | Proportion of patients with positive culture |
| --- | --- | --- |
| Escherichia coli | 4 | 0.018 |
| Coagulase-negative Staphylococcus spp. | 3 | 0.014 |
| Pseudomonas aeruginosa | 3 | 0.014 |
| Acinetobacter baumanii | 2 | 0.0090 |
| Streptococcus pneumoniae | 2 | 0.0090 |
| Candida spp. | 2 | 0.0090 |
| Staphylococcus aureus | 2 | 0.0090 |
| Klebsiella pneumoniae | 2 | 0.0090 |
| Enterococcus spp. | 1 | 0.0045 |
| Stenotrophomonas maltophilia | 1 | 0.0045 |
| Streptococcus spp. | 1 | 0.0045 |

**Supplementary Table 3:** Reported sources of superinfection among YF patients at any point during hospitalization. Infections described at multiple sites are reported individually.

| Source | Count |
| --- | --- |
| Pulmonary | 19 |
| Unspecified | 11 |
| Abdominal | 6 |
| Skin and soft tissue | 3 |
| Esophageal candidiasis | 1 |
| Biliary | 1 |
| STI | 1 |
| Endocarditis | 1 |
| Urinary | 1 |
